# Perceptions of primary healthcare providers for screening and management of mental health disorders in India: a qualitative study

**DOI:** 10.3389/fpubh.2024.1446606

**Published:** 2024-10-04

**Authors:** Ramesh Kumar Sangwan, Darshana Kansara, Santosh Matoria, Haider Ali, Mukti Khetan, Vishal Singh, Mahendra Thakor, Ramesh Kumar Huda, Bontha V. Babu

**Affiliations:** National Institute for Implementation Research on Non-Communicable Diseases, Jodhpur, Rajasthan, India

**Keywords:** mental health, primary healthcare, perceptions, challenges, training

## Abstract

**Introduction:**

Individuals experiencing mental health disorders encounter numerous challenges while accessing mental healthcare services. Despite the inclusion of mental health in the primary healthcare system, screening and managing mental health disorders remain significantly overlooked. Therefore, there is a need to understand the perceptions of healthcare providers in primary care settings, identify the challenges faced, and gather suggestions for effective mental healthcare delivery.

**Methods:**

The present qualitative study was conducted in 13 primary healthcare facilities in the Jodhpur district of Rajasthan, India, from 2023 to 2024 among 25 primary healthcare providers. Semi-structured interview guides were developed for each category of primary healthcare provider, including Medical Officers, Community Health Officers, and General Nurse Midwives, Auxiliary Nurse Midwives and in-depth interviews were recorded, transcribed, and thematically analyzed using codes and sub-codes.

**Results:**

Results are summarized under the themes: (i) Navigating Mental Healthcare in Primary Healthcare Facilities, (ii) Challenges and Barriers in Mental Healthcare Delivery, (iii) Recommendation for Enhancing Mental Healthcare Services, and (iv) Approaches for Comprehensive Capacity Building Training and Module and further findings of each theme are presented under various sub-themes.

**Conclusion:**

The findings suggest that despite a perceived competence in providing mental health services, there were substantial levels of negative attitudes towards mental health disorders among participants and provide insights for policymakers and healthcare professionals to develop targeted interventions and improve mental healthcare delivery at primary care facilities.

## Introduction

Globally, one in every eight individuals is affected by a mental disorder. These disorders are characterized by substantial disruptions in cognitive function, emotional regulation, or behaviour. Mental health disorders emerged as the primary contributor to disability, affecting one in every 6 years of healthy living (1). A wide range of mental disorders exists, each with its unique challenges. Health systems worldwide have not sufficiently addressed the needs of individuals with mental disorders and remain critically under-resourced. Although there are effective prevention and treatment strategies available, the majority of people with mental disorders lack access to appropriate care. The disparity between the demand for treatment and its availability is vast across the globe, and when care is provided, it is often of inadequate quality (2).

According to the World Health Organization, India shoulders a significant burden of mental health disorders, amounting to 2,443 disability-adjusted life years (DALYs) per 100,000 individuals. The economic consequences of mental health disorders in India are staggering, with estimated losses totalling USD 1.03 trillion between 2012 and 2030 (3). In the state of Rajasthan, the occurrence rates of common mental disorders stand at 10.1%, alcohol use disorders at 2.6%, and depressive disorders at 2.7%. However, there exists a significant treatment gap for these conditions, with 87.4% for common mental disorders, 85.6% for alcohol use disorders, and 93.9% for depressive disorders (4).

The 2016 National Mental Health Survey in India revealed that common mental disorders, which include depressive and anxiety disorders, affected 5.1% of the population. Alarmingly, the treatment gap for these disorders was reported at a staggering 80.4% (5). Individuals experiencing mental health disorders encounter numerous challenges when attempting to access mental healthcare services. These challenges are particularly heightened among marginalized communities or those residing in remote areas, resulting in irregular utilization of such services. The resulting treatment gap may stem from disparities in the allocation of mental health resources and inconsistencies in the execution of mental health policies (6, 7).

The need for integrating mental health with primary healthcare services is considerable. Firstly, integration guarantees timely and uninterrupted access to mental healthcare for the entire population at the onset of disorders. Secondly, receiving treatment at primary healthcare facilities enhances the prospects of improved health outcomes, potential full recovery, and sustained social integration for individuals (8). The Indian government is actively enhancing mental healthcare services at the primary healthcare level. Mental healthcare services are now included in the Comprehensive Primary Health Care package under the *Ayushman Bharat -Health and Wellness Centre* Scheme. Operational guidelines for addressing *Mental, Neurological, and Substance Use Disorders (MNS)* at Health and Wellness Centres have been issued as part of the Ayushman Bharat initiative (9).

Despite the inclusion of mental healthcare in primary healthcare and in the national program to provide comprehensive primary healthcare services in the country, screening and managing mental health disorders remain significantly overlooked at the primary care level. Therefore, it is necessary to understand healthcare providers’ perceptions in primary healthcare settings to identify the challenges faced and gather suggestions for effective mental healthcare delivery. It will contribute to developing a more effective module for training primary healthcare providers in managing mental health disorders in primary healthcare settings.

## Study area and participants

The study is a mixed method approach conducted in 13 Primary Health Centres, Community Health Centres, Health and Wellness Centres and Satellite hospitals across different blocks of the Jodhpur district in the state of Rajasthan in India from 2023 to 2024. The interviews were conducted in the district’s rural and urban areas. A total of 25 Primary Healthcare Providers were selected for this study: six Medical Officers, seven Community Health Officers, six General Nurse Midwives, and six Auxiliary Nurse Midwives for interviews. Out of these, three Medical Officers, seven Community Health Officers, two General Nurse Midwives, and two Auxiliary Nurse Midwives have received prior training under different organizations, respectively. Semi-structured interview guides were prepared for each category of primary healthcare providers, including Medical Officers (who have 4.5 years academic duration +1-year internship in Bachelor of Medicine and Bachelor of Surgery), General Nurse Midwives (who have a 3.5-year diploma in general nurse-midwifery or a 4-year bachelor degree in nursing), and Community Health Officers (who have a 4-year bachelor degree in nursing or 4.5-year academic duration +1-year internship in Bachelor of Ayurvedic Medicine and Surgery) and Auxiliary Nurse Midwives (who have a 2-year diploma in auxiliary nurse midwifery).

This study is part of a project aimed at enhancing the capacity of primary healthcare providers in the screening and management of common mental disorders in primary care settings. It will help equip healthcare providers with the necessary skills and knowledge to address mental health challenges at the primary care level. The specific objective of this qualitative study was to gain insights into the perceptions of healthcare providers working in primary healthcare settings in Rajasthan, India. These insights are required to develop training material on screening and managing common mental ailments in primary healthcare.

Data were collected through qualitative, in-depth interviews among different categories of healthcare providers (10). The interview covered various aspects of mental healthcare in primary care, exploring their perception, challenges in delivering mental healthcare and training needs for their effective roles in primary healthcare facilities. Interviews were conducted in the state’s local language, Hindi.

## Interview guide

The interview guide for medical officers, community health officers, general nurse midwives, and auxiliary nurse midwives includes questions to understand their roles in the primary healthcare system, particularly mental healthcare. These guides were developed based on the existing primary mental healthcare guidelines and literature on primary mental healthcare. They were further refined through the brainstorming of researchers and public health and mental health experts. The guides covered the perceptions of mental health, training experiences, and preparedness for screening and diagnosing mental health disorders. The guide also explores their management and treatment practices, the resources available at the primary care level, and their challenges, including stigma and infrastructure barriers. Additionally, it seeks their observations on patient outcomes and suggestions for improving mental health training and mental healthcare delivery. The interview guide with designed questions has been added as Supplementary material (interview guide) at the end of the paper. The interviews were conducted by the research team with trained public health and social science professionals after receiving study-specific in-house training in qualitative research. This training ensured consistency and accuracy throughout the data collection and analysis process.

## Data analysis

A grounded theory approach was employed to analyze the qualitative data. This method was chosen due to its flexibility and suitability for exploratory research contexts where existing knowledge is limited (11). All interviews were transcribed by listening to the audio recordings while cross-checking the notes taken during the sessions. The transcripts were subsequently translated into English, with some being back-translated to ensure linguistic accuracy and fidelity. We adhered to standard guidelines for the transcription and translation of qualitative data throughout the process (12, 13). Data coding was done using a deductive approach. Themes were identified, and thematic content analysis was performed (14). The qualitative data analysis included these six stages, i.e., familiarization of the data, initial coding of the dataset, searching for themes, reviewing themes, defining, naming themes, and producing the report. The study research team did the analysis. Lastly, the themes and quotes from the participants were presented with anonymous identification (13, 15).

## Results

This study used thematic analysis to refine the overarching themes and sub-themes by systematically analysing the codes and subcodes extracted from the transcripts. Four main themes and fourteen sub-themes were finalized through this iterative process (Table 1).

**Table 1:** Themes and corresponding sub-themes.

Theme	Sub theme
Navigating mental healthcare in primary healthcare facilities	Overview of primary healthcare responsibilities
	Experience and understanding of mental health
	Treatment, management, and referral process
	Mental healthcare resources and facilities at the primary level
Challenges and barriers in mental healthcare delivery	Screening and diagnosing challenges
	Treatment and management challenges
	Resources and facilities challenges
Recommendation for enhancing mental healthcare services	Implementation of training program and infrastructure development
	Community engagement and mental health promotion
	Professional support and collaboration
	Continued training and retraining approaches
Approaches for comprehensive capacity building training and module	Suggestion for training mode/type
	Approaches for standardized content and protocols in module
	Retraining, follow-up and broader outreach

### Theme 1: navigating mental healthcare in primary health centres

This theme explores the current scenario of mental healthcare delivery at primary healthcare centres. It highlights the comprehensive responsibilities of healthcare providers in addressing mental health disorders, their understanding and experiences with mental health, as well as the treatment, management, and referral processes involved. Additionally, it examines the availability and adequacy of mental healthcare resources and facilities at the primary level.

#### Overview of primary healthcare responsibilities

This sub-theme highlights healthcare providers’ broad responsibilities in delivering mental healthcare at primary healthcare centres. It encompasses tasks such as patient identification, assessment, and initial management of mental health disorders.

The responses provide insights into healthcare professionals’ diverse roles and responsibilities at primary and community health centres. Participants highlighted various aspects such as routine Outpatient department management, community-based approaches, early diagnosis, and timely patient referrals. They emphasized the importance of training and organizing programs for primary healthcare workers like ASHAs (Accredited Social Health Activists who are community health workers in India) and Auxiliary Nurse Midwives to ensure effective field operations and community engagement. Additionally, the participants discussed their roles in patient interaction, administrative tasks, and multipurpose works aimed at maintaining hygiene and cleanliness at healthcare facilities.

*“Overall work, such as routine Outpatient department, comes here, patients suffering from seasonal diseases like a cough in winter, and patients related to mental health come here after getting a prescription from outside and medicines from here.” (Medical Officer from rural health facility).*

*“Our work is mostly to see the patients coming to the Outpatient department. We have to do the patients coming to us online and check the Ayushman card. Apart from this, we have to do field visits and interact with the patients.” (Community Health Officer from rural health facility).*

*“We go to the field, make Ayushman cards* (health insurance card)*, make Aadhar cards* (national identification card)*, see the patient, refer the patient, we do such work.”(Community Health Officer from rural health facility).*

*“Our biggest job is to care for mother and child and all this work of Ayushman Bharat and ABHA Card* (Ayushman Bharat Health Account- linking their medical records across healthcare facilities) *remains ours.” (Auxiliary Nurse Midwifery from rural health facility).*

*“Because there are cases, then we know this. We check whether he has taken this medicine properly or not. If the doctor says that a report should be made, we record it, when the medicine was taken, when it was not, and when it started.” (General Nurse Midwifery from rural health facility).*

*“Yes, Accredited Social Health Activists workers and Auxiliary Nurse Midwifery go to the field, our thing is that whatever diseases occur and from time to time as our normal programs are going on.” (Medical Officer from rural health facility).*

*“Our role is to do early diagnosis and timely referral of patients, which is my main work in the PHC* (Primary Health Centre)*; the rest of the national programs going on are to be implemented in our PHC.” (Medical Officer from rural health facility).*

*“I order whatever indent medicines are short, I send reports, I get field surveys done.” (Medical Officer from rural health facility).*

*“Multipurpose work is a relief, hygienic, maintenance, and cleanliness.” (Medical Officer from rural health facility).*

#### Experience and understanding of mental health

Here, the focus is on understanding the perspectives and experiences of healthcare providers regarding mental health. It discusses their level of awareness, knowledge, and perceptions of mental health disorders, as well as the challenges they face in addressing them.

The theme revealed a multifaceted understanding of mental health disorders, treatment approaches, and prevalent challenges. Participants emphasized the interconnectedness of mental and physical health, noting that mental well-being affects various aspects of life, including work engagement and social interactions. There was recognition of the importance of addressing mental health concerns at the primary care level, with an emphasis on counselling and community support. Moreover, participants highlighted the prevalence of common mental disorders such as depression, anxiety, and substance abuse, particularly among younger and middle-aged individuals from low- to middle-income groups. While diagnostic capabilities were acknowledged, limitations in resources and facilities underscored the need for referral protocols and higher-centre interventions. Additionally, the study shed light on societal challenges, including stigma and misconceptions, emphasizing the imperative for awareness and education initiatives within communities.

*“The thing about health is that it depends a lot on a person’s mental health. All functions of the body are related to mental health. One who has proper mental health, be it his interest in his work or his eco-social life, mental health affects everything. It is important for a person to be mentally fit more than physically fit because mental health is correlated with other systems.” (Medical Officer from rural health facility).*

*“And there are beedies* (hand-rolled cigarettes)*, cigarettes and gutka (chewing tobacco). When we do screening there, we ask whether he drinks or not, whether he chews gutkha or not, we ask all this during the screening.” (General Nurse Midwifery from urban health facility).*

*“Mentally, it can be due to family problems, financial issues, or socio-economic factors, which have different elements.”(Community Health Officer from rural health facility).*

*“Regarding mental health, I would like to say that any condition or disease which hinders the physical functioning of a person or his family life or personal life, we will define that as our mental health. Age-wise, it is more in younger and middle age group, almost 25 to 40–50, and more in this range. By gender, it is higher among men, and by income, it is higher among low- and middle-income groups.” (Medical Officer from rural health facility).*

*“In this area, you will see mostly drug addicts, whether it is alcohol or smoking; most people are addicted to drugs.” (Community Health Officer from rural health facility).*

*“That’s what madam, those who suffer from depression mostly consume drugs and if anyone does, if he has any mental illness, we explain it to him or refer him to a doctor at PHC* (Primary Health Centre)*.” (Auxiliary Nurse Midwifery from rural health facility).*

*“So, there is nothing like that; in today’s time, there is a cure for everything.” (Medical Officer from rural health facility).*

*“The most important thing is to talk and tell the mental patient that this is not a long-term illness. In today’s time, there is treatment, and it is cured not so much by medicines as behaviour therapy.” (Medical Officer from rural health facility).*

#### Treatment, management, and referral process

This sub-theme explores the approaches taken in treating and managing mental health disorders at the primary level. It also looks into the processes involved in referring patients to specialized care facilities when necessary and ensuring continuity of care.

The study findings reveal key insights into the treatment, management, and referral processes for common mental disorders at the primary healthcare level. Medical officers recognize the importance of targeted treatment for early common mental disorders signs, offering medications like Alprazolam, anti-depressant medications, and benzodiazepines. Challenges in integrating mental healthcare include delayed family responses, inadequate formal mental health data management systems, and patient non-adherence to treatment regimens. These issues hinder effective treatment and recovery. However, there is a growing emphasis on integrating physical and mental healthcare, incorporating non-pharmacological interventions, and providing continuous social support. Community health officers are crucial in identifying patients, offering guidance, making referrals, and monitoring medication adherence. Despite these efforts, improvements are needed in data management, patient education, and community engagement. Insights from field observations reveal that mental health data is often combined with routine Outpatient department visits without separate tracking, and treatment at the primary level includes managing symptoms with available medications. There is a lack of a formal referral system for mental health, and some health workers may overlook mental health disorders, focusing instead on more immediate concerns like vaccinations. To enhance recovery-oriented care, it is essential to improve family engagement, understand and address barriers to treatment adherence, and strengthen community support. This approach aims to improve health outcomes, support full recovery, and facilitate sustained social integration for individuals.

*“There are different categories in this; there are some initial symptoms, like if a small child misbehaves or there is a sudden behaviour change, then we have treatment for that level.” (Medical Officer from rural health facility).*

*“There is no such special mental health data; it is in routine Outpatient department only, like a diabetic patient is coming, he is going for follow up, blood pressure is going for follow up, and some mental health and tuberculosis patients are common. There is nothing separate.” (Medical Officer from rural health facility).*

*“Yes, for example, take a case of proper depression, a case of proper panic disorder, then we manage it at our level, give benzodiazepine, propranolol as much as we know and guide him at that level.” (Medical Officer from rural health facility).*

*“Whatever anti-depressant is there, like Alprazolam, Benzodiazepam, all these are there.” (Medical Officer from rural health facility).*

*“When people come to us, we cannot refer them ourselves; we have to send them to the medical officer, and then they refer them when they have had their council. But right now, we do not have any system for mental health.” (Community Health Officer from rural health facility).*

*“As per our guideline, at the PHC* (Primary Health Centre) *level, mental health patient comes, after giving him primary treatment, we refer him to the higher centre in a proper manner through the referral system.” (General Nurse Midwifery from rural health facility).*

*“Mental health is like torture from all sides. We do not look much into those who do not have good mental balance and have little brain. We only vaccinate children.” (Auxiliary Nurse Midwifery rural health facility).*

*“Someone becomes irritable, you do not check properly, you do not treat properly, we understand that he is mentally ill.” (Auxiliary Nurse Midwifery from rural health facility).*

*“We refer him to the doctor here for treatment.” (Auxiliary Nurse Midwifery from rural health facility).*

#### Mental healthcare resources and facilities at primary level

This sub-theme examines the availability and adequacy of resources and facilities for mental healthcare delivery at the primary health level. It investigates the challenges stemming from resource shortages and infrastructure limitations that impact service provision.

Health workers’ responses highlight various aspects of mental healthcare resources and facilities at the primary level. Medical officers focus on direct referrals for severe cases and monitor medication adherence and treatment progress assessment through documentation. The routine camps referred to here are mental health awareness and screening camps crucial for early detection and intervention. Despite challenges, such as the absence of these routine mental health camps and limited interventions thus far, essential medications like Alprazolam, Clonazepam, and Imipramine are available. However, there is a pressing need for improved guidelines and standardized tools, as current practices do not adequately incorporate these elements. Current practices do not adequately incorporate these elements, particularly those related to mental health screening and treatment. The presence of only one Accredited Social Health Activist and insufficient data on mental health disorders highlights the necessity for enhanced community engagement. While resources like posters promoting mental health awareness are available, there is significant potential for improvement in organizing camps and raising awareness about tobacco and drug bans.

*“Medicines are available as per standard at our PHC* (Primary Health Centre)*. One is Alprazolam, Clonazepam, Imipramine, and Cetirizine; all these medicines are available.” (Medical Officer from rural health facility).*

*“The facility is just the medicine; the rest is the consultation part or referral part from our side.” (Medical Officer rural health facility).*

*“No, there is nothing like that, we have never used any kind of guideline or tool.” (Medical Officer from rural health facility).*

*“There is no separate camping.” (Medical Officer rural health facility).*

*“There is such a program and we want to run this program and increase health awareness by setting up camps, then he gives you permission and then we can manage it at our level.” (Medical Officer from rural health facility).*

*“Well, severe ones are referred directly.” (Medical Officer rural health facility).*

### Theme 2: challenges and barriers in mental healthcare delivery

Focusing on the challenges faced in mental healthcare delivery, this theme highlights challenges in accurately screening and diagnosing mental health disorders, treating and managing patients effectively, and accessing sufficient resources and facilities. It sheds light on the barriers hindering the early identification and assessment of mental health disorders and explores obstacles related to resource shortages and inadequate infrastructure.

#### Screening and diagnosing challenges

This sub-theme highlights the difficulties in properly screening and diagnosing mental health disorders within primary healthcare settings. It explores the barriers hindering early identification, assessment, and strategies to overcome these challenges.

Responses highlight various challenges faced by healthcare providers in diagnosing mental health disorders at primary healthcare centres. Participants emphasized the difficulty in accurately diagnosing psychiatric disorders due to the similarity of symptoms and the absence of definitive diagnostic tests, relying primarily on patient history for diagnosis. Furthermore, challenges arise from patients’ reluctance to disclose mental illness, fear of social stigma, and resistance to accepting psychiatric labels. Limited attention and resources contribute to difficulties in adequately screening and diagnosing patients, compounded by communication barriers and misinterpreting symptoms. Additionally, the community’s lack of awareness and misconceptions about mental health further hinder early identification and treatment.

*“Because the biggest thing about psychiatry is that we are not able to diagnose ourselves so easily, all the symptoms seem to be similar, sometimes it will be of depression, sometimes there will be something else, so we cannot diagnose it so easily.” (Medical Officer from rural health facility).*

*“The main thing is that the patient has his motive; the patient does not give his details. We have to ask about it, but indirectly, he tries to hide it somewhere, and there is a little social dilemma. If by chance we are diagnosed with a mental disorder, will it send us further to the higher Centre or will some negative feelings come towards us among our social people, hence this is the main problem.” (Medical Officer from rural health facility).*

*“Such patients come to our centre who have mental problems, so looking at them, it is clear that they have mental problems, but they do not know; they think that we live in the village, and somehow, we are in the village. Perhaps the wind caught us or some kind of shadow fell on us, or I stepped in the wrong place.” (Community Health Officer from rural health facility).*

#### Treatment and management challenges

This sub-theme focuses on the obstacles encountered in treating and managing mental health disorders. It addresses issues such as medication adherence, therapy provision, and patient follow-up within primary healthcare settings.

Healthcare providers highlighted various challenges in treating and managing mental health disorders at primary healthcare centres. One significant issue identified was medication adherence challenges, where patients sometimes resist taking medication, leading to conflicts and non-compliance. Creative solutions, such as mixing medication with juice, were suggested to address this resistance. Maintaining consistent treatment was also emphasized, with healthcare providers stressing the importance of following prescribed dosages to ensure effectiveness.

*“No, we tell the person accompanying the patient that sometimes if the patient quarrels, then he has to be convinced and given the medicine; sometimes it happens that he does not take the medicine and starts fighting, then through some other means like medicines have to be given by mixing them in juice or through some other medium.” (Medical Officer from rural health facility).*

*“The problem is that people who suffer from mental health are told that there is no such thing as this medicine will last for your entire life.” (General Nurse Midwifery from rural health facility).*

Resistance and conflict were observed in severe mental health cases, with patients often not complying with treatment recommendations and engaging in confrontations with their families. Stigma and social isolation were identified as significant barriers to follow-up care, with patients and families often avoiding seeking further treatment due to societal attitudes toward mental health. Furthermore, challenges in providing adequate time for counselling and follow-up were noted, indicating limitations in addressing patients’ suffering and understanding their needs due to time constraints. Additionally, challenges in mental health treatment protocols were identified, with healthcare providers expressing a lack of defined protocols and guidelines for alternative drugs, leading to limitations in treatment options.

*“Follow-up is a big challenge for us; we are not able to create a network for that; how do we follow the patients and how do they monitor them regularly.” (Medical Officer from rural health facility).*

*“If I tell this to anyone then they tell us that we do not have any such disease; they themselves say that we are fine as if they know that if they have the disease, they do not want to go and the people of the village So they are not able to go, they want us to get all the things around us.” (Community Health Officer from rural health facility).*

*“No, there is nothing like that, we have never used any kind of guideline or tool.” (Medical Officer from rural health facility).*

#### Resources and facilities challenges

This section emphasizes the challenges arising from resource shortages and inadequate mental healthcare delivery facilities at the primary health level. It investigates funding, staffing, and infrastructure barriers that impact service provision and patient outcomes.

The response highlights the challenges in providing comprehensive mental healthcare. Participants emphasized the absence of resources and facilities beyond medication, including specialized counsellors and dedicated counselling arrangements. The lack of specialized training for healthcare workers in mental health diagnosis was also noted. Furthermore, there was a significant gap in awareness and resources for mental health education, such as a shortage of modules and awareness campaigns. These findings underscore the need for increased resources, specialized training, and awareness campaigns to bridge mental healthcare delivery gaps.

*“There is no resource available here. We do not have any special counsellor here. Normally our sir does the counselling. There is no separate arrangement for counselling.” (General Nurse Midwifery from rural health facility).*

*“There is no separate facility available for mental health programs here.” (Medical Officer from rural health facility).*

The insights from other respondents further emphasize significant challenges in accessing mental healthcare. Participants expressed frustration over the lack of medications available. The absence of specific protocols and workshops addressing mental healthcare exacerbates the issue, indicating a critical gap in healthcare training and infrastructure.

*“No, there is nothing like that, we have never used any kind of guideline or tool.” (Medical Officer from rural health facility).*

*“This is the biggest problem, so first of all, a separate cell should be formed.” (Medical Officer from urban health facility).*

### Theme 3: recommendations for enhancing mental healthcare services

This theme offers recommendations for enhancing mental healthcare services at primary healthcare centres. It suggests implementing comprehensive training programs for healthcare providers and infrastructure improvements to facilitate better service delivery. Furthermore, it advocates for community engagement initiatives, professional support networks, and continued training to ensure healthcare providers remain up-to-date with best practices.

#### Implementation training program and infrastructure development

This sub-theme suggests strategies for implementing comprehensive training programs for healthcare providers and infrastructure improvements to enhance mental healthcare services at primary healthcare centres.

The theme responses highlight the essential insights provided by participants regarding the need for comprehensive mental health training programs and infrastructure development. Participants emphasized the necessity of government-led screening programs for population-wide assessment, particularly at the primary healthcare level. They stressed the importance of incorporating mental health training into programs like child mental health programs. They emphasized the need for community-based mental health counselling centres to provide support and awareness at the grassroots level. Additionally, there were suggestions for enhancing the effectiveness of training programs by incorporating implementation approaches and addressing challenges faced by healthcare professionals in attending training sessions.

*“If we look at the PHC* (Primary Health Centre) *level, whether it is the Government of India or the State Government if they do screening and programs from time to time, then it will be known that out of the population of this village, how many patients are coming for psychiatric treatment.” (Medical Officer from rural health facility).*

*“Separate arrangements should be made for counselling, like once a year, wherever the government may open its centre from the village level.” (Medical Officer from rural health facility).*

*“It is exactly so, sir; there should be a separate room for mentally ill patients so that we can talk to them comfortably and understand them.” (Community Health Officer from rural health facility).*

*“The things for which we do not have the facilities should be added; we have taken so much training, and, obviously, we have also studied a lot at the undergraduate level. Psychiatry was a different subject, but the implementation part is the most special thing.” (Medical Officer from rural health facility).*

#### Community engagement and mental health promotion

Focusing on community involvement, this sub-theme advocates for initiatives aimed at raising awareness, reducing stigma, and promoting mental health within communities served by primary healthcare centres.

The sub-theme of “Community Engagement and Mental Health Promotion” underscores the need to address stigma, misconception, and lack of awareness surrounding mental health within communities. Participants highlighted the pervasive stigma associated with psychiatric medicines and the misconception regarding their impact on fertility, which hampers female patients’ access to treatment. Additionally, there is a lack of awareness about conditions like obsessive-compulsive disorder, with limited recognition of diverse symptoms and a dearth of educational modules to address this gap.

*“Today you hold a mental health awareness camp, and I should also post a WhatsApp message in the community that if I send a message to those who have such problems, 10–20 will definitely come, but I should have that skill and knowledge so that I can address them.” (Medical Officer from rural health facility).*

The above quote highlights the interconnectedness of awareness efforts, community engagement, and the need for skill development among healthcare workers to address mental health concerns effectively. To connect these ideas, it’s important to note that mental health camps not only serve to raise awareness but also provide a platform for early identification and management of mental health disorders within the community.

*“The first thing is to acknowledge the stigma in the community.” (Medical Officer from rural health facility).*

*“The first thing we should do is to provide training at the PHC* (Primary Health Care) *level and also run awareness programs in the community so that there is awareness about mental health.” (Community Health Officer from rural health facility).*

*“The problem is that what to talk about in the community, we do not know, what is there in this is the community site, there is no module on how you will explain obsessive-compulsive disorder to the patient.” (Medical Officer from rural health facility).*

*“Firstly, it is very important to increase awareness among the people about its importance; this can be taken care of at the PHC* (Primary Health Centre)*, CHC* (Community Health Centre), *primary level so that they can come to the place and solve their problems. And if seen at the first level, then at the first level, they should first come to the hospital so that their treatment can start right there.” (General Nurse Midwifery from urban health facility).*

*“Why is that, then first of all, the patient’s demand should be fulfilled, for that we need top-level facilities, drug supply should be maintained well.” (Medical Officer from rural health facility).*

*“Today you hold a mental health awareness camp, and I should also post a WhatsApp message in the community that if I send a message to those who have such problems, 10–20 will definitely come, but I should have that skill and knowledge so that I can address them.” (Medical Officer from rural health facility).*

*“If a woman is made aware about everything from time to time, she will definitely become alert.” (Auxiliary Nurse Midwifery from rural health facility).*

*“Just talk about the disease, just broadcast what is going on now in the community, dengue is going on, you have talked about dengue, if any such patient or child is found, then he should be motivated and told.” (Medical Officer from urban health facility).*

*“At the community level, I would like to say that there is depression, anxiety, and substance abuse, about which if people are given information, or if it is spread at the community health level, then many psychiatric issues can be avoided.” (Medical Officer from urban health facility).*

#### Professional support and collaboration

The emphasis here is on fostering professional support networks and collaboration between healthcare providers, mental health specialists, and community resources to improve mental healthcare delivery.

The sub-theme of emphasizes the necessity of engaging with stakeholders like government offices, Non-Governmental Organizations (NGOs), and experienced professionals to enhance mental health services. Participants advocated for authorization and support from higher authorities, such as the Chief Minister’s office, to effectively conduct awareness campaigns and health camps. They stressed the importance of having MD (Doctor in Medicine) psychiatrists or addiction specialists present at camps for early intervention and treatment initiation. Suggestions were made for establishing special centres or deploying special doctors dedicated to mental healthcare. Mentorship programs were highlighted as crucial for skill enhancement among mental health professionals, with proposals for incentivizing such initiatives. Collaboration with NGOs was essential for spreading awareness and leveraging resources, emphasising facility development and network expansion to ensure comprehensive treatment approaches.

*“If we go to the Chief Minister’s office and talk to the chief minister and tell him that, sir, there is such a program, and we want to run this program and increase health awareness by setting up camps, then he gives you permission, and then we can manage it at our level.” (Medical Officer from rural health facility).*

*“Their visit is necessary; if there are camps, then orthopaedics and ophthalmologists come, MD medicine also comes, similarly MD psychiatric should also come so that if there is any patient, he can be screened at the ground level and if the medicine is available, then his treatment can be started there or someone can motivate the family member about the health centre, and in rural areas it seems that the value of a big doctor is a little higher.” (Medical Officer from rural health facility).*

*“There should be special centres or special doctors for them where they can go and get their treatment.” (Medical Officer from rural health facility).*

*“Some people from that village are also nominated for the medical team. That someone please help us. When we came to the village, we also explained it to his family. Well, this is a disease.” (General Nurse Midwifery from rural health facility).*

*“Yes, of course, like he should have a mentor, every trained individual psychiatrist must have a matter who is an experienced psychiatrist.” (Medical Officer from rural health facility).*

*“And we should take the help of NGOs like I myself am an Art of Living volunteer, okay, we work a lot on mental health.” (Medical Officer from rural health facility).*

*“Yes, we can absolutely collaborate, because if we go to secondary prevention identification and treatment, then it will not make sense.” (Medical Officer from rural health facility).*

#### Continued training and retraining approaches

This sub-theme recommends ongoing training and retraining initiatives to ensure healthcare providers remain updated on best practices and developments in mental healthcare.

The sub-theme of underscores the importance of ongoing training and retraining to enhance mental health services. Participants emphasized the need for specialized training at the Community Health Centre level to ensure effective service delivery. They advocated for continuous monitoring and updates to keep abreast of evolving trends in mental healthcare. While rejecting the necessity for frequent retraining, they suggested a more sustainable approach, such as annual retraining. They proposed an extended training duration of 6 months to a year for comprehensive understanding and implementation. Additionally, participants highlighted the significance of case studies in understanding mental illness, recommending a timeframe of 6 months for studying mental health disorders and emphasizing the importance of counselling and behaviour management training for primary healthcare workers to promote patient understanding and effective treatment.

*“For this, I think that if we are training a person every time, that is not going to help. Well, first of all, we should make a little change that for this we need to train at CHC* (Community Health Centre) *specially.” (Medical Officer from rural health facility).*

*“We should keep the provision of the Medical Officer, that this is a trend in mental health, this mental health will be properly looked after sir, whatever work is done, they have to see it properly, and do the same later, if any updates come then share, treat.” (Medical Officer from rural health facility).*

*“There should be proper training, there should be a screening tool so that we can find out by talking.” (Community Health Officer from a rural health facility).*

*“There is no need for 3 months, yes six months is good, rest if it is annual then it will work, and there is no problem.” (Medical Officer from rural health facility).*

*“All our staff should be trained. How should we detect the patient, and how should we behave with him to treat him well? To reach where he can, training is necessary.” (General Nurse Midwifery from rural health facility).*

*“As a psychiatrist, this is the thing, so if we proceed in this manner, then the training can be done in 6 months, or even if it is for a year, we can keep it.” (Medical Officer from rural health facility).*

### Theme 4: approaches for comprehensive capacity building training and module

This theme highlights comprehensive capacity-building strategies and discusses various training modalities, including online courses and workshops, to enhance healthcare providers’ skills and knowledge. It recommends standardized content and protocols for training modules and emphasizes the importance of continuous follow-up and feedback mechanisms. Additionally, it suggests integrating practical approaches such as case studies and role-playing exercises into training programs for better learning outcomes.

#### Suggestion for training mode/type

This sub-theme explores different training modalities such as online courses, workshops, and simulations to enhance the skills and knowledge of healthcare providers in delivering mental healthcare.

The responses regarding comprehensive capacity-building training and module approaches emphasize the preference for offline training formats due to their perceived advantages. Medical officers highlighted active participant engagement and note-taking opportunities and suggested a training duration of 4 to 5 h for effectiveness. Offline counselling was favoured for its ability to observe non-verbal cues. Respondents proposed various training methodologies such as blended approaches, practical training, and a one-week duration, emphasizing the importance of exposure, reflection, and comprehensive subject matter coverage.

*“Offline training is good because when people have questions, they can be answered quickly and visually. The second thing is that if you are getting some questions and some inputs are coming, then you are also able to make your notes.” (Medical Officer from rural health facility).*

*“I think 4 h of training is enough; no one can concentrate for more than 4 h,” and we should include more practical training in the training rather than giving lecture theory; what should be assured him is that the counselling is being targeted, CBT* (Cognitive Behavioral Therapy) *response therapy, he should know whatever counselling comes.” (Medical Officer from rural health facility).*

*“Or show it through video only then we can know what is a thing and what is not so that we can know what behaviour a person behaves like it is understood in the movie, is not it? If we have that base, we can tell this thing is prevalent in the community.” (Community Health Officer from rural health facility).*

The insights provided by community health officers and auxiliary nurse midwives further underline the importance of training duration, format, and content. Suggestions included online/offline options for 3–4 h, offline weekly training, and a focus on practical knowledge alongside theoretical lectures. Additionally, recommendations for shorter training periods, concise content delivery, and inclusion of video lectures and practical demonstrations were highlighted.

*“Our training is for 7 days, but it is better if we teach for 2–3 days.” (Auxiliary Nurse Midwifery from rural health facility).*

*“It should be offline and of one-week duration, and the training should be such that it is properly usable for us as to how we can assess or guide them.” (Community Health Officer from rural health facility).*

*“Training classes should be in the form of lectures which can also include video lectures. Classes can be both offline and online. Practical knowledge is also necessary.” (Community Health Officer from a rural health facility).*

*“Offline, because even if you face some issue offline, you can clear it manually so it does not go to the next level. Similarly, you can do it online, but if you ask theoretically, offline is better.” (General Nurse Midwifery from urban health facility).*

*“Which should include study material, PDF, videos,* etc.*, and practical knowledge. There should also be booklet notes,* etc.*, which will be helpful in revising.” (Community Health Officer from rural health facility).*

#### Approaches for standardized content and protocols in module

The focus is on standardizing content and protocols for mental health training modules to ensure consistency and adherence to evidence-based practices.

The responses from healthcare professionals highlight several key suggestions for enhancing comprehensive capacity-building training and module development in mental healthcare. They emphasize the importance of practical utility and accessibility in training content, advocating for content that is easy to understand and apply. Participants stressed the need for regular updates and refreshment courses to keep healthcare providers informed about changes in treatment protocols and medications. Moreover, the significance of patient-centric training in hospitals is underscored, focusing on comprehensive follow-up processes, effective communication skills, proper counselling, and establishing a counselling system to ensure optimal patient care. Additionally, there are calls for offline counselling therapy, explanations of common mental disorders, and training by psychiatrists from basics to ensure effective training delivery. The suggestions emphasize the importance of tailored training content, practical approaches, and patient-centred care to enhance mental health services effectively.

*“There should be content about those useful routines, what utilization is, and how mental health affects routine health. The content should be such that everyone can understand it, it should be easy to read, and it should be easy to apply.” (Medical Officer from rural health facility).*

*“Now that the treatment guidelines have changed, there should be a refreshment course as per the current guidelines. This means that what are the current guidelines? Have any norms changed, or has any new medication been introduced? How will you implement that thing? There have been many changes, like Lithium, which is not considered very safe, and we cannot give it.” (Medical Officer from rural health facility).*

*“We should get some such training that the psychiatrist himself will give us training and will explain to us from zero to whom we should refer further and whom we can treat at our level.” (Community Health Officer from rural health facility).*

#### Retraining, follow-up and broader outreach

This theme emphasizes the need for structured follow-up and periodic retraining to evaluate and reinforce mental health training. It also highlights expanding training to a broader audience and engaging communities to enhance mental health awareness. Participants highlighted key strategies to improve mental health training by emphasizing the importance of follow-up, periodic retraining, and broader participation. A systematic follow-up process was recommended to assess the effectiveness of the training over time, allowing for reflection on its outcomes only after participants had applied their skills in real-world settings. Retraining and counselling were also suggested at 3 to 6 months to reinforce learning and provide ongoing support. To maximize the impact of mental health awareness, participants urged the extension of the training to a wider audience, including health workers at the primary health centre and community health centre levels, and the promotion of community outreach through awareness campaigns at the schools and village levels. These approaches may strengthen mental healthcare by ensuring consistent evaluation, skill reinforcement, and widespread community engagement.

*“We will talk about them only after the follow-up training and what results have been achieved so far.”*

*“It will take time. A short time is a minimum of 3 months; the maximum could be 6 months, so our retraining and counselling should be back in this duration.”*

*“My suggestion regarding training would be that the training can be made better in such a way that if training is given at PHC* (Primary Health Centre) *and CHC* (Community Health Centre) *level, then more people can join and participate in it and apart from the doctors, there are other people too. If there are medical health workers, add them, teach them in this training, hold camps in schools, hold camps till village level, only then can they bring awareness in mental health through posters,* etc.*”*

## Discussion

For optimal effectiveness and efficiency, mental health in primary care should be integrated with a comprehensive network of services across various levels of care, complemented by broader enhancements to the healthcare system. Incorporating mental health services into primary care represents the most effective strategy for bridging the treatment disparity and guaranteeing individuals receive the necessary mental health services (8, 16, 17). Analyzing the participants’ stories validated the original system components and uncovered varying connections between them, suggesting potential areas for further exploration (18). The current study unveils a lack of knowledge and hands-on proficiency among healthcare providers operating in primary healthcare institutions and within the community in India. The study results underscore several key findings regarding challenges and recommendations for enhancing mental healthcare at the primary care level.

Theme one focuses on mental healthcare in primary care facilities. It includes the responsibilities of healthcare providers, their overall exposure and understanding of mental health and mechanisms for treatment, management, referral and available resources and facilities. This section highlights the importance of patient management, community-based approaches, early diagnosis, timely referrals, and training for primary healthcare workers. These findings align with a study where participants emphasized their roles and identified functions like clinical services, community resources connections, health education and coaching, patient interaction, administrative duties, and maintaining hygiene at healthcare facilities (19, 20). A review underscored the lack of formalized and standardized mental health training and its monitoring for community-level workers (21). A study finding observed that almost one-third of the participants had not received any training in providing mental healthcare, highlighting gaps in knowledge and skills necessary for effective mental healthcare delivery (22). It holds significant policy and clinical relevance for India, where there is a shortage of trained mental health professionals, suggesting that trained healthcare workers can help fill the gap in mental health diagnosis and services (23). The present study also observed that various participants do not have any prior training experience associated with mental health, which is aligned with the findings from a study conducted in Kashmir during initial interactions with the Community Health Workers at the start of the training program; they noted participants had limited exposure to training programs focused on mental health (24).

Our second theme highlights the challenges and barriers related to screening and diagnosing, treatment and management, and resources and facilities for mental healthcare within primary care settings. Participants face challenges such as the similarity of conditions due to symptom similarities, patient reluctance, the community’s lack of awareness, misconceptions associated, medication adherence, non-compliance and resource limitations. These factors not only hamper early identification and assessment but also impact treatment adherence and follow-up care (25–27). Widespread stigma and the fear of being judged by others can influence young people to seek support for their mental health (28). Our findings support a study by Cowan et al. that showed that doctors providing primary healthcare in rural Karnataka, India, observed substantial levels of negative attitudes towards mental health disorders among participants (29). Treatment and management challenges included medication adherence issues, stigma-related barriers and a lack of defined protocols (30, 31). Moreover, resource shortages and inadequate facilities hindered comprehensive mental healthcare delivery, emphasizing the need for specialized training and infrastructure improvements. Stigma and social isolation exacerbate these challenges, as patients and families may avoid seeking further treatment due to societal attitudes towards mental health. Findings from our study are congruent with another study from Kafczyk T et al., which identifies challenges such as poor public governance, lack of awareness among policymakers, and existing policies that may overly medicalize mental health problems, hindering the effective delivery of mental healthcare (32).

Theme third of the study emphasized effective mental healthcare delivery at the primary care level; recommendations included implementing offline training programs, standardizing content and protocols, facilitating continuous follow-up and feedback, engaging in community awareness campaigns, enhancing infrastructure and resources, integrating mental health into existing programs, and fostering collaboration between healthcare providers and stakeholders (27, 30). The emphasis on offline training formats highlights the need for interactive sessions that allow healthcare providers to engage actively and learn through practical methods such as case studies (26). By implementing these recommendations, primary care facilities can enhance their capacity to provide comprehensive care. Integrating practical training methods and standardized protocols can equip healthcare providers with the necessary skills and knowledge to screen, diagnose, treat, and manage mental health disorders effectively (25). The results of our study align with another study, which found that individuals who received health facility-based care and community counselling experienced better health outcomes than those who only received care from health facilities (33). Additionally, community engagement initiatives can help bridge gaps in awareness and reduce the stigma surrounding mental health, ultimately leading to better outcomes for individuals and communities affected by mental disorders (27, 30). These findings provide insights for policymakers and healthcare professionals to develop targeted interventions and improve mental healthcare delivery at primary care facilities. The Ayushman Bharat initiative’s Mental, Neurological and Substance Use care training program holds significant promise in addressing the challenges and barriers identified in the study’s discussion regarding mental healthcare delivery (25). A study by Kilbourne et al. recommends assessing mental health outcomes more regularly, and measurement-based care must be integrated into existing technologies and embedded within the treatment culture and healthcare system (34). Given the global mental health crisis, prioritizing research on mental health delivery systems has become essential (18).

The last theme comprised approaches for comprehensive capacity-building training and modules, including suggestions for the future training mode/type, content and protocols, and further follow-up and feedback. Given the range of capacity-building methods already in place, offline training formats may be preferred, as they are often more practical and feasible. Similar findings were observed in a meta-analysis, which states that in settings where various capacity-building methods are already available, internet-based interventions may not provide an advantage over existing, more practical approaches, considering the significant investment needed to establish online learning platforms and supporting infrastructure (35). Our study emphasized the importance of practical utility and accessibility in training content, which is similar to the study findings, which shows that the training should incorporate a validated assessment tool tailored to the needs of community-level workers, along with a structured resource guide or manual in local languages to support them after training (21). Standardized training modules are crucial in ensuring consistency and efficacy in mental healthcare delivery by incorporating evidence-based practices and regular updates (30, 36).

The study’s strength lies in its comprehensive exploration of various facets of mental healthcare delivery at the primary care level (25). Through qualitative analysis, the study effectively captures the perspectives of healthcare providers, shedding light on challenges and barriers faced in screening, diagnosing, treating, and managing mental health disorders (26). Furthermore, the study offers valuable recommendations for enhancing mental healthcare services, including strategies for capacity building, standardized training modules, and community engagement initiatives. These insights provide a solid foundation for policymakers, healthcare administrators, and practitioners to develop targeted interventions and improve mental healthcare delivery.

There are some limitations to consider when analysing the findings of the study. The reliance on qualitative data may introduce subjective biases in data interpretation, potentially affecting the validity and reliability of the study’s conclusion. Furthermore, the study primarily captures the perspectives of healthcare providers, potentially overlooking the experiences and needs of individuals with lived experience of mental health disorders (30). The data were not based on probability sampling, so the findings cannot be generalized. Addressing these limitations through broader sampling strategies, mixed-methods approaches, and participatory research methodologies could enhance the robustness and applicability of future studies in this area.

## Conclusion

Integrating mental healthcare services into primary care is vital for addressing treatment disparities. Challenges such as accurate diagnosis and resource limitations hinder early intervention. For effective mental healthcare delivery, offline training programs, standardized protocols, and community engagement are essential. By enhancing healthcare provider skills and reducing stigma, primary care facilities can better support individuals with mental health needs. While this study offers valuable insights, addressing limitations like subjective biases and broader perspectives is crucial for future research. Ultimately, targeted interventions informed by these findings can significantly improve public health outcomes covering mental health.

## Data Availability

The raw data supporting the conclusions of this article will be made available by the authors, without undue reservation.
