# Corrigendum: Perceptions of primary healthcare providers for screening and management of mental health disorders in India: a qualitative study

**DOI:** 10.3389/fpubh.2024.1519716

**Published:** 2024-12-02

**Authors:** Ramesh Kumar Sangwan, Darshana Kansara, Santosh Matoria, Haider Ali, Mukti Khetan, Vishal Singh, Mahendra Thakor, Ramesh Kumar Huda, Bontha V. Babu

**Affiliations:** National Institute for Implementation Research on Non-Communicable Diseases, Jodhpur, Rajasthan, India

**Keywords:** mental health, primary healthcare, perceptions, challenges, training

In the published article, reference 20 was incorrectly written as:

20. Sangwan RK (2014) Health rights of patients and medical ethics of doctors.

The corrected reference details appear below:

20. Sangwan RK. Health rights of patients and medical ethics of doctors. *J. Soc. Res*. (2012) 8:34–43.

The authors apologize for this error and state that this does not change the scientific conclusions of the article in any way. The original article has been updated.

