# Supplementary material for: Perceptions of primary healthcare providers for screening and management of mental health disorders in India: a qualitative study
Source: Front Public Health. 2024 Oct 4;12:1446606. doi: 10.3389/fpubh.2024.1446606 (PMC11486692; doi:10.3389/fpubh.2024.1446606)
Supplement: Supplementary file 1 [file Table_1.DOCX]

**Interview Guide**

**Medical Officers**

**Introduction**

- Brief introduction of the interviewer and the participant.
- Role in the primary health care system.
- Years of experience and key responsibilities.

**Perceptions**

- Views on mental health care and its importance at the primary care level.
- Frequency of dealing with patients experiencing anxiety, depression, and substance use disorders.

**Training and Preparedness**

- Psychiatry training received during graduation and its effectiveness.
- Training programs attended and mode of delivery (online, offline, hybrid).
- Challenges in identifying mental health disorders (anxiety, depression, substance use).

**Management and Treatment**

- Care for patients with mental health disorders.
- Availability of medications, prescription practices, non-pharmacological interventions (e.g., counselling), and referrals to specialists.
- Patient follow-up, compliance, and any challenges faced.

**Infrastructure and Resources**

- Availability of resources and tools for mental health care at the primary level.
- Barriers or gaps in mental health infrastructure.

**General Observations and Stigma**

- Inferiority complex in patients and stigma in the community (social, religious, and workplace-related).

**Monitoring and Support**

- Monitoring service delivery and managing data.
- Suggestions for improving services and mental health training.

**General Nursing Midwives and Community Health Officers**

**Introduction**

- Brief introduction of the interviewer and the participant.
- Role in the primary health care system and years of experience.

**Perceptions and Understanding**

- Importance of mental health.
- Understanding of common mental health disorders (e.g., anxiety, depression) and substance use.

**Training**

- Mental health training received.

**Screening and Communication**

- Methods for communicating with and screening mental health patients.
- Challenges in screening and support from senior staff.

**Risk Assessment**

- Management of high-risk patients and ensuring their safety.

**Awareness and Stigma**

- Involvement in community awareness programs and training of community health workers.
- Observations of individual and community stigma.

**Support and Suggestions**

- Feedback on improving mental health care skills and training for frontline workers.

**Auxiliary Nurse Midwives**

**Introduction**

- Brief introduction of the interviewer and the participant.
- Role and responsibilities in primary health care.

**Perceptions and Understanding**

- Understanding of mental health, common disorders, and vulnerable populations.

**Training**

- Mental health training received and its application in patient care.

**Screening and Communication**

- Screening processes, challenges in identifying mental health patients, and support from senior staff.

**Risk Assessment**

- Managing high-risk patients at the community level and referral practices.

**Awareness and Stigma**

- Role in mental health awareness programs at the primary care level and sub-centre.
- Observations of stigma at both individual and community levels.

**Support and Suggestions**

- Suggestions for overcoming screening and awareness challenges and improving training programs and manuals for community health workers.
